# Immunotactoid Glomerulopathy Masquerading as Heart Failure

**DOI:** 10.7759/cureus.63687

**Published:** 2024-07-02

**Authors:** Kabeer Ali, Avni Agrawal, Abhinav Karan, Christopher Marsalisi, Melville C O'Brien, Shiguang Liu, Vishal Jaikaransingh

**Affiliations:** 1 Internal Medicine, University of Florida College of Medicine, Jacksonville, USA; 2 Pathology and Laboratory Medicine, University of Florida College of Medicine, Jacksonville, USA; 3 Nephrology, University of Florida College of Medicine, Jacksonville, USA

**Keywords:** tactoid body, fibrillar glomerulopathy, light chain deposition disease, immunotactoid glomerulopathy, immunotactoid

## Abstract

Immunotactoid glomerulopathy (ITG) is a rare form of glomerular disease. It is characterized by organized, dense immunoglobulin deposits in the glomerulus, impairing glomerular function and filtration. The prognosis tends to be poor, and the majority of patients develop end-stage renal disease (ESRD). Here, we present a case of a young male with no prior medical history who presented with anasarca. His presentation was initially thought to be due to a new diagnosis of heart failure with a decreased ejection fraction. However, significant proteinuria led to a diagnosis of ITG.

## Introduction

Glomerulopathy encompasses a wide variety of diseases and is characterized by immune-mediated damage. As classifying these diseases can be complex, they can be further broken down into amyloid-mediated and non-amyloid-mediated. Non-amyloid-mediated diseases include immunotactoid glomerulopathy (ITG) and fibrillary glomerulonephropathy [1]. ITG is characterized by organized, dense immunoglobulin deposits in the glomerulus. The deposits are larger, over 30 mm, and usually arranged in parallel or stacked. The overall prevalence of ITG is very low, being present in only 0.5% to 1.4% of kidney biopsies [2]. As this is a rare disease entity, it is not usually considered as a differential in patients presenting with anasarca. In addition, the finding of a low ejection fraction was unexpected.

## Case presentation

**Figure 1:**
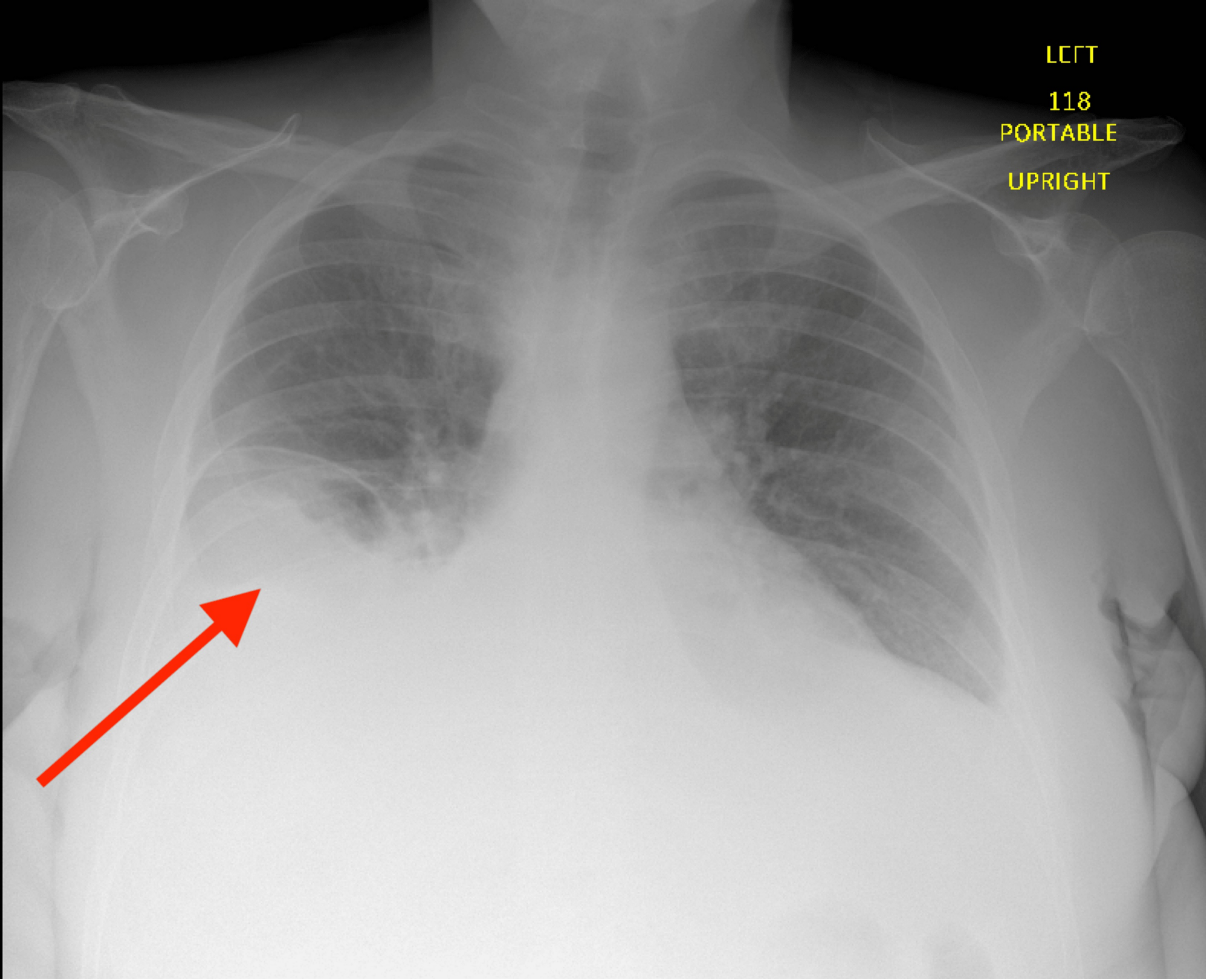
Chest radiograph showing bilateral pleural effusions, right greater than left (arrow)

A 45-year-old Caucasian male with no past medical history presented with orthopnea and worsening generalized body swelling. He reported that his anasarca began seven months before his presentation, beginning with his legs and progressing to his arms and abdomen. In the emergency department, he met the criteria for a hypertensive emergency with an elevated cardiac troponin to 338 ng/L and elevated serum creatinine to 5.9 mg/dL. The electrocardiogram showed no ischemic changes, while the chest radiograph revealed evidence of vascular congestion and bilateral pleural effusions (Figure 1). Physical examination was significant for elevated jugular venous pressure, normal heart sounds, pitting edema from the lower extremities extending into the sacrum and hips bilaterally, bilateral upper extremity edema, abdominal distention, and a positive fluid wave.

He was started on a nitroglycerin infusion at 10 mg/min and a furosemide infusion at 15 mg/h for blood pressure control and to promote diuresis. His hospital course was complicated by acute kidney injury. Laboratory investigations were significant for hypoalbuminemia to 2.2 mg/dL. Urinalysis was positive for microscopic hematuria and greater than 500 mg/dL protein. The urine protein/creatinine ratio was 7.53, and the microalbumin/creatinine ratio was 4202 mg/g of creatinine. The N-terminal prohormone of brain natriuretic peptide is greater than 70,000 pg/mL. Serum protein electrophoresis showed no evidence of a monoclonal paraprotein. The 24-hour urine protein was 2385 mg protein in 24 hours (Table 1). Transthoracic echocardiogram showed bi-atrial dilation with a 40%-45% reduced ejection fraction. Blood pressure was controlled, and a transition to oral nifedipine 60 mg daily and hydralazine 100 mg twice a day was made. Despite high doses of diuretic with torsemide 100 mg daily, the patient had minimal urine output and refractory volume overload, leading to the initiation of hemodialysis.

**Table 1:** Laboratory values at presentation with reference ranges for comparison

Laboratory investigation	Patient value	Reference range
Serum troponin	368 ng/L	<22 ng/L
Serum creatinine	5.9 mg/dL	0.6-1.2 mg/dL
Albumin	2.2 mg/dL	3.8-49 mg/dL
Urine RBC	6 RBC per high-power field	0-5 RBC per high-power field
Urine protein	292 mg/dL	Negative
Urine protein/creatinine ratio	7.53	<0.2
Urine microalbumin/creatinine ratio	4202	0-29
24-hour urine protein	2385 mg/24 h	<150 mg/24 h
N-terminal prohormone of brain natriuretic peptide	>70000 pg/mL	0-125 pg/mL

**Figure 2:**
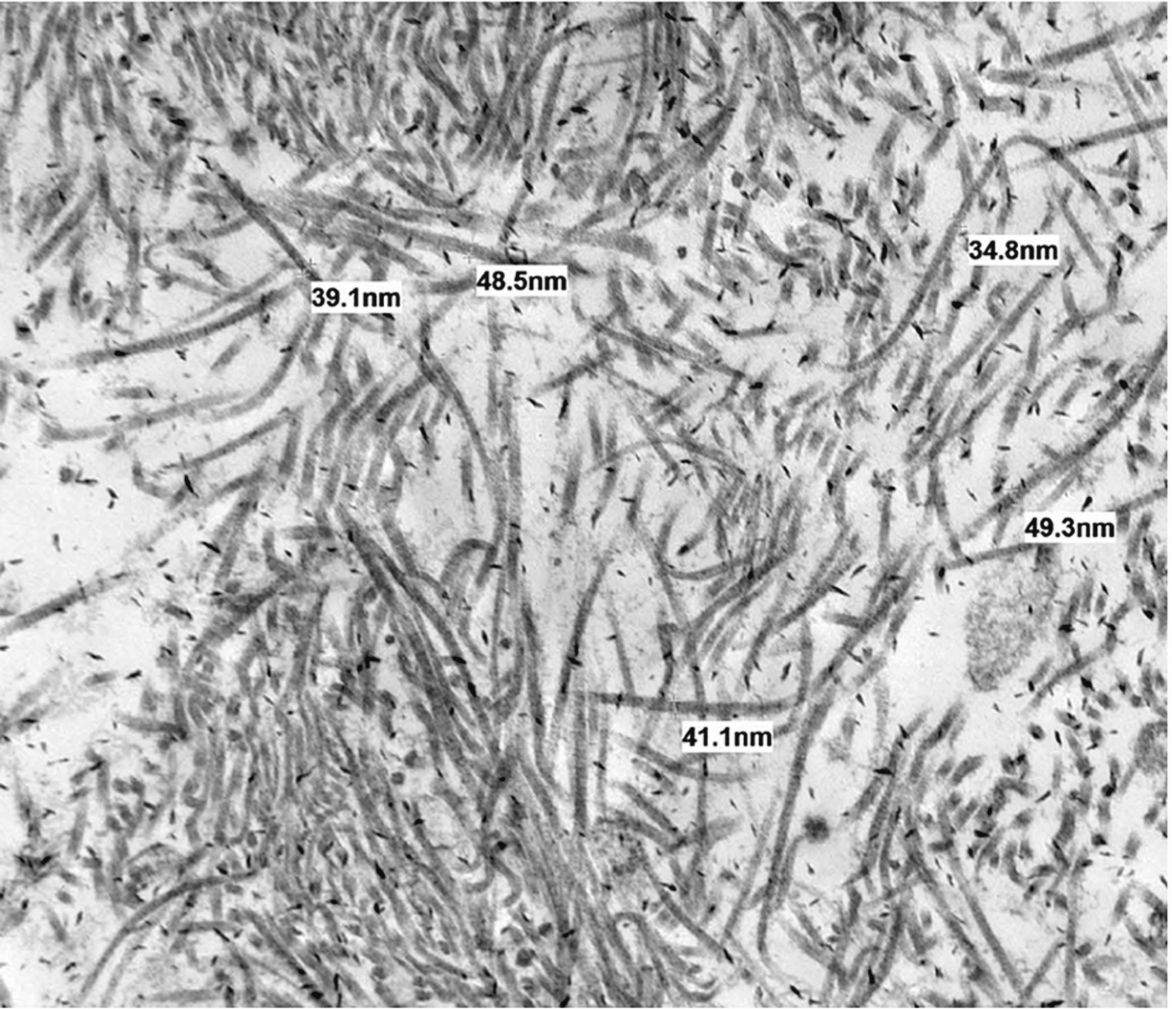
Electron microscopy at 6000 times magnification showing immunotactoid deposition with a large diameter of microtubule deposits (average of 41 nm)

**Figure 3:**
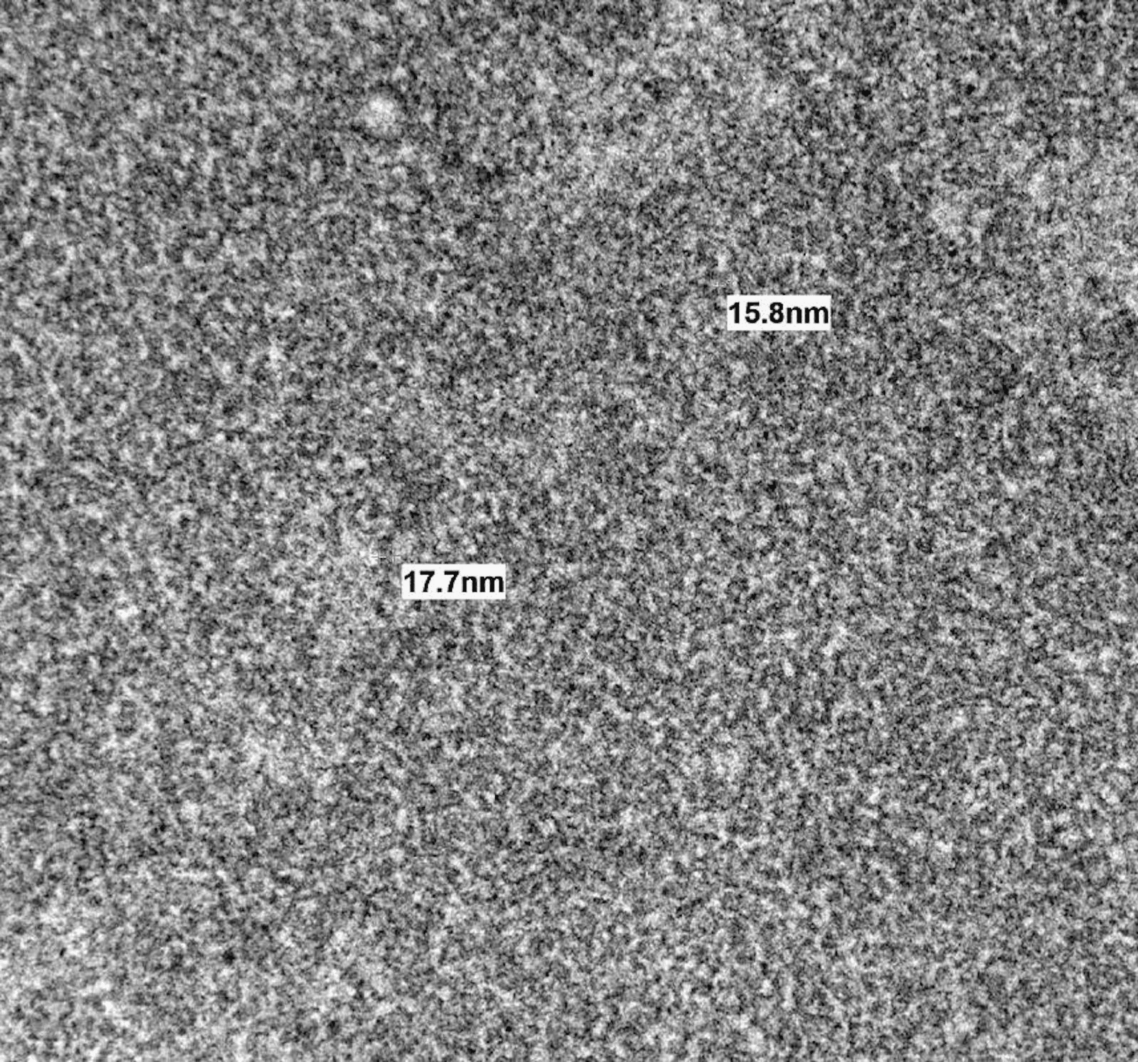
Electron microscopy image highlighting further immunotactoid deposits of smaller diameter

Due to the large degree of proteinuria, a kidney biopsy was performed, examining 26 adequately oriented glomeruli. A 65% of glomeruli appeared globally sclerotic, with segmental sclerosis and glomerular basement membrane thickening in the remainder. Plasma insudation was present, but no endocapillary proliferation, crescent, or neutrophil infiltration was identified. There was severe interstitial fibrosis and tubular atrophy. Immunofluorescence studies showed no specific immunoglobulins, complement or kappa, lambda light-chain deposits. Notably, Congo red staining was negative for amyloidosis. Electron microscopy was consistent with organized microtubular deposits in a mesangial region with an average diameter of 41 nm extending to the capillary loops (Figures 2, 3). Microtubules within this range meet the criteria for cryoglobulinemic and ITG. Cryoglobulinemia was excluded clinically, with negative serum cryoglobulin, a negative hepatitis panel, and normal complement levels. In addition, the patient did not have Meltzer's triad of purpura, weakness, and arthralgias. This favored a diagnosis of primary ITG, and unfortunately, the patient remained dependent on hemodialysis.

## Discussion

ITG was first described in 1980 when it was noted that one patient’s glomerular deposits were reminiscent of the linear crystallization of hemoglobin S that forms elongated tactoids in RBCs during sickle cell crisis [3]. Primary ITG, in which patients present with proteinuria, may have hematuria, and the majority of patients have underlying hypertension or diabetes. It is an infrequent phenomenon, with some data suggesting that it occurs in less than 0.1% of native kidney biopsies [4]. Retrospective data looking at cases of ITG over 20 years showed all patients displayed proteinuria. A case series of 73 patients with ITG reported that 76% of these patients had nephrotic range proteinuria, and nephrotic syndrome was seen in 57% of these patients [4]. Korbet et al. reported that as much as 40% of patients diagnosed with ITG progressed to end-stage renal disease (ESRD) [5]. This data demonstrates that while this disease entity is rare, it has a severe impact on renal function with a high prevalence of ESRD.

Common causes of anasarca include cardiac failure, liver failure, lymphatic system disruption, and renal failure. Sometimes, there may be overlap between two or more of these in a single presentation, such as light-chain amyloidosis causing both congestive cardiac failure and nephrotic range proteinuria [6]. In this scenario, fibrils of protein subunits deposit in several organs, such as the heart and kidney, in beta-pleated sheet configuration. While amyloidosis exists on a spectrum of organized light-to heavy-chain deposition diseases, immunotactoid is non-amyloid-mediated [7]. In this case, excluding other common non-amyloid glomerular-deposition diseases was essential.

A study examining morphological features of fibrillary glomerulonephritis versus ITG showed the microtubule fibril diameter was, on average, 43.2+/-10.3 nm compared to fibrillary glomerulonephritis, which was 14.0+/-0.5 nm [1]. This is consistent with our patient's diameter, where the average diameter of their deposits was 41 nm. Cryoglobulinemic nephritis has a similar range of fibril diameter but is still usually smaller than ITG. A key differentiating factor is that while cryoglobulinemic deposits are rarely seen as subepithelial, immunotactoid deposits are often distributed in a subepithelial pattern [8]. The overall clinical picture, such as in our patient with a negative hepatitis panel, serum cryoglobulin, normal complement levels, and lack of characteristic physical examination findings, would point away from cryoglobulinemia. An interesting point to note is that our patient’s serum protein electrophoresis did not show evidence of a monoclonal protein. There is an association between ITG and hematologic malignancy, with one study showing up to 38% of patients diagnosed with ITG had a co-existing hematologic malignancy [9].

Currently, there is no proven therapy for ITG; current favored therapies include steroid monotherapy or immunosuppressive agents with or without steroids. A study of 48 patients compared treatment modalities of those with ITG [10]. In this study, five patients received steroid monotherapy and 34 received immunosuppressive therapy with or without steroids. Complete remission was seen in one patient in the steroid monotherapy group and 14 in the immunosuppressive group. In these groups, nine patients progressed to ESRD. Primary ITG remains a sporadic disease without clear guidelines for treatment. In our patient, the only presenting symptom was progressive volume overload. With the patient's reduced ejection fraction, elevated n-terminal-pro brain natriuretic peptide, and dyspnea, it was initially presumed he was in an acute heart failure exacerbation. However, after discovering the patient's nephrotic range proteinuria and low albumin, underlying renal disease was favored instead of cardiac involvement. After multiple sessions of hemodialysis, the patient’s volume status improved. Currently, there are no guidelines or clear evidence on the use of immunosuppressive agents or steroid use. Case reports have reported corticosteroids being used to treat ITG with a positive response, but no firm guidelines currently exist on this [11]. Our patient’s kidney biopsy showed severe scarring, as demonstrated by global glomerulosclerosis in 65% of sampled glomeruli, severe interstitial fibrosis, and tubular atrophy. This would not have been altered by immunosuppressive therapy. As such, the risk of such treatment was thought to outweigh the benefits. An outcome study comparing ITG to fibrillary glomerulonephropathy shows inferior survival in patients with ITG on dialysis [12]. However, prognosis and disease recurrence rates were comparable in both groups following renal allografts. Primary ITG remains rare with a low overall incidence, so prompt diagnosis is needed.

## Conclusions

Primary ITG has an overall low incidence with an unfavorable prognosis leading to renal failure. The patient initially presented with concern for acute decompensated heart failure; however, after the discovery of significant proteinuria, this led to a kidney biopsy and diagnosis of ITG. All causes of proteinuria should be excluded, and kidney biopsy remains the gold standard today, where biopsies are safer, and more treatment options become available. ITG remains relatively rare, so this paper aims to inform clinicians about early recognition and improved patient outcomes.
